# Pro-Myogenic Environment Promoted by the Synergistic Effect of Conductive Polymer Nanocomposites Combined with Extracellular Zinc Ions

**DOI:** 10.3390/biology11121706

**Published:** 2022-11-25

**Authors:** José Luis Aparicio-Collado, José Molina-Mateo, Constantino Torregrosa Cabanilles, Ana Vidaurre, Beatriz Salesa, Ángel Serrano-Aroca, Roser Sabater i Serra

**Affiliations:** 1 Centre for Biomaterials and Tissue Engineering, Universitat Politècnica de València, 46022 Valencia, Spain; 2 Biomedical Research Networking Centre in Bioengineering, Biomaterials and Nanomedicine (CIBER-BBN), 46022 Valencia, Spain; 3 Biomaterials and Bioengineering Lab, Centro de Investigación Traslacional San Alberto Magno, Universidad Católica de Valencia San Vicente Mártir, 46001 Valencia, Spain; 4 Department of Electrical Engineering, Universitat Politècnica de València, 46022 Valencia, Spain

**Keywords:** pro-myogenic environment, bioactive cell-material interface, conductive polymer nanocomposites, zinc ions, carbon nanomaterials, graphene, skeletal muscle tissue engineering

## Abstract

**Simple Summary:**

Musculoskeletal tissue can self-regenerate after injury, however, this self-renewal capacity is limited in degenerative diseases or volumetric muscle loss. Tissue engineering strategies involving biomaterials, cells, and bioactive agents have emerged as a tool to regenerate damaged skeletal muscle. The role of biomaterials is not only to provide structural support for tissue regeneration but also to include some biophysical and biochemical cues that enhance cell proliferation and differentiation into different tissues. In this context, electrochemical cues are essential for myofiber motility and myoblast differentiation. Here, we engineered electrically conductive nanocomposites, which will promote bioactivity in the form of intrinsic surface conductivity, close to that of human skeletal muscle tissue. In addition, extracellular zinc ions were incorporated in the cell microenvironment as a myogenic factor. We show that the combination of both approaches acts synergically generating enhanced cell microenvironments that promote myogenesis.

**Abstract:**

A new strategy based on the combination of electrically conductive polymer nanocomposites and extracellular Zn^2+^ ions as a myogenic factor was developed to assess its ability to synergically stimulate myogenic cell response. The conductive nanocomposite was prepared with a polymeric matrix and a small amount of graphene (G) nanosheets (0.7% *wt*/*wt*) as conductive filler to produce an electrically conductive surface. The nanocomposites’ surface electrical conductivity presented values in the range of human skeletal muscle tissue. The biological evaluation of the cell environment created by the combination of the conductive surface and extracellular Zn^2+^ ions showed no cytotoxicity and good cell adhesion (murine C2C12 myoblasts). Amazingly, the combined strategy, cell–material interface with conductive properties and Zn bioactive ions, was found to have a pronounced synergistic effect on myoblast proliferation and the early stages of differentiation. The ratio of differentiated myoblasts cultured on the conductive nanocomposites with extracellular Zn^2+^ ions added in the differentiation medium (serum-deprived medium) was enhanced by more than 170% over that of non-conductive surfaces (only the polymeric matrix), and more than 120% over both conductive substrates (without extracellular Zn^2+^ ions) and non-conductive substrates with extracellular Zn^2+^. This synergistic effect was also found to increase myotube density, myotube area and diameter, and multinucleated myotube formation. MyoD-1 gene expression was also enhanced, indicating the positive effect in the early stages of myogenic differentiation. These results demonstrate the great potential of this combined strategy, which stands outs for its simplicity and robustness, for skeletal muscle tissue engineering applications.

## 1. Introduction

The musculoskeletal system, responsible for the movement and support of any vertebrate model, is a complex tissue composed of aligned multinucleated and contractile cells known as myofibers [1]. Musculoskeletal tissue can self-regenerate after injury in a coordinated process by recruiting immature and quiescent satellite cells [2]. These cells proliferate, fuse, and differentiate into myoblasts that reorganize themselves to form, first, myotubes, and then myofibers. However, this self-renewal capacity is limited in degenerative diseases or volumetric muscle loss, resulting in fibrosis and muscular impairment [3]. Although different treatments (cell and gene therapies, autotransplants, allotransplants, or xenotransplants) are able to enhance muscle regeneration, none of them seems to successfully overcome the issue due to immunogenicity, poor cell survival or lack of stability [4,5]. Tissue engineering (TE) has emerged as a tool to regenerate damaged or lost tissue by providing cells with an environment that mimics the extracellular matrix (ECM) so that they can proliferate and differentiate into new tissue [6,7,8]. Skeletal muscle TE has been shown to stimulate survival and differentiation of myoblasts towards tissue regeneration both in vitro and in vivo [5,9,10].

Functional muscle tissue contracts in response to the electrical signals produced by motoneurons. Electrochemical cues are essential for both myofiber motility and myoblast differentiation in a still unknown mechanism that seems to be related to the induction of the calcineurin pathway mediated by ion flow through the cell membrane [11,12,13]. In this context, electroconductive scaffolds based on polymeric matrices with incorporated conductive polymers, such as poly(3,4-ethylenedioxythiophene), polypyrrole (PPy), polyaniline or carbon nanomaterials (CNMs), have been developed for muscle regeneration [14,15,16,17,18,19]. Graphene nanomaterials are a family of CNMs that include few-layer graphene, graphene nanosheets, graphene quantum dots, graphene oxide (GO), and reduced-graphene oxide (rGO) [20]. Graphene (G) is a polycyclic aromatic hydrocarbon with potential applications in TE thanks to its excellent mechanical properties, high conductivity, biocompatibility, and nanoscale surface roughness able to match cell receptors and mimic the ECM [21]. It has been used in combination with different polymers, such as chitosan, gellan gum, or polycaprolactone (PCL) to prepare composite biomaterials that induced muscle regeneration [1,22,23]. However, its cellular toxicity and immunological effects are both concerns that must be taken into account when CNMs are proposed for biomedical applications [24,25]. It has been reported that CNM biocompatibility is highly dependent on the concentration and processing techniques used to obtain the biomaterial [26,27]. Cardiac, neural, and skeletal muscle tissues have electrical stimuli-responsible properties. Thus, the conductive properties of in vitro models are an important tool in the reproduction of in vivo environments, which have electrical properties related to the diffusion of electrical charges within the ECM [28]. Conductive biomaterials, such as graphene-based biomaterials, even in the absence of electrical stimulation, have been shown to stimulate cell response in skeletal muscle cell differentiation [29,30]. In a recent study, we engineered electroactive nanohybrid hydrogels based on sodium alginate/PCL semi-IPNs with rGO nanosheets, which possess conductive properties in the range of muscle tissue [18]. Myoblast adhesion and myogenic differentiation were greatly enhanced with the incorporation of 2% of conductive rGO nanosheets.

The role of biomaterials is not only to provide structural support for tissue regeneration, but also to include some biophysical and biochemical cues that enhance cell proliferation and/or differentiation into different tissues. The use of growth factors, such as vascular endothelial growth factor (VEGF) and insulin-like growth factor (IGF), have been reported to stimulate myoblast proliferation, differentiation, and myotube formation [31,32], although they can also generate problems related to alteration in cell homeostasis and risk of cancer [33,34]. Bioactive molecules, including bio-ceramics and therapeutic inorganic ions, have been investigated for tissue regeneration due to their ability to stimulate tissue regeneration without the disadvantages of growth factors [35,36]. Promising results have been obtained with biometals in the field of regenerative medicine, due mainly to their stability and ability to induce cell response. Trace elements, such as copper, iron, or zinc, are considered therapeutic ions [37,38,39]. In particular, zinc biometal, both as inorganic ion (Zn^2+^) and zinc oxide nanoparticles, is being studied for its role as myogenic factor, since it has been shown to induce cell proliferation, differentiation, and migration, accelerating in vitro muscle formation [39,40]. Zinc homeostasis is essential for skeletal muscle tissue [41] and regulates different metabolic signaling pathways, such as the phosphoinositide 3′-kinase (PI3K)/Akt pathway, directly involved in skeletal myogenesis [42,43]. Extracellular Zn^2+^ stimulates the PI3K/Akt pathway through stimulating the Zip7 transporter in murine C2C12 cells, considerably raising proliferation and myogenic differentiation [40].

Although both electrically conductive surfaces and extracellular zinc ions have been reported to stimulate myoblast differentiation (as we showed in previous studies [18,40]), to the best of our knowledge, there is no evidence in the literature on their possible synergistic effects in myogenesis when used together.

In this study, we engineered electrically conductive nanocomposites based on a polymeric matrix with incorporated G nanosheets, which will enhance bioactivity in the form of intrinsic surface conductivity, close to that of human skeletal muscle tissue. In addition, extracellular Zn^2+^ ions were incorporated in the cell microenvironment (culture medium) as myogenic factor. The concentration of Zn^2+^ ions in the culture medium (40 µM) was based on previous results [40], which showed that this specific concentration induced a positive effect on myogenesis stimulation.

We have chosen a polymer matrix based on PCL, the well-known biomaterial approved by the Food and Drug Administration (FDA), widely used in biomedical applications due to its excellent biocompatibility, physicochemical properties, and good processability [44]. TE approaches have used PCL extensively as a cell substrate to enhance the regeneration of different bone, cartilage, skin, nerve, dental, or skeletal muscle tissue [45,46,47] with different configurations, such as flat substrates [47], nanofibers [48], interconnected channel networks [49], or 3D printed supports [50]. Highly conductive CNMs, particularly G and rGO nanoparticles, are good candidates for engineering electrically conductive nanocomposites, although the conductive properties of G are higher than those of rGO. Thus, a very small percentage of G nanosheets was selected to avoid potential cytotoxicity effects, and to reduce costs, since CNMs are still expensive materials.

The aim of this work was thus to test a new strategy for skeletal muscle TE based on the combination of conductive polymer nanocomposites (electrically conductive surface) together with a specific extracellular zinc (Zn^2+^) concentration on myoblast adhesion, proliferation, and the early stages of myogenic differentiation. We hypothesized that the combination of both approaches would act synergically to generate enhanced cell microenvironments, promote myogenesis, and so become a simple, effective, and low-cost tool applicable to skeletal muscle TE.

## 2. Experimental

### 2.1. Materials and Reagents

Polycaprolactone (MW: 43–50 kDa) was supplied by Polysciences, Warrington, USA (Reference 19561). Graphene powder in the form of nanosheets (Reference 900561), and Zinc Chloride (ZnCl_2_) (Reference Z0152) were purchased from Merk. Tetrahydrofuran (THF) was bought from Scharlab, Spain (Reference TE02282500). All the reagents were used as received.

### 2.2. Preparation of Conductive Cell Substrates

Polycaprolactone was dissolved at a 3% *wt*/*wt* concentration in THF under constant stirring at 25 °C for two hours and transferred into Petri dishes. Neat PCL films (reference sample) were obtained after 72 h of solvent evaporation at room temperature (RT). The protocol for preparing cell substrates with a conductive surface was adapted from previous studies [18,51]. Briefly, 14 mg of G nanosheets (0.7% *wt*/*wt* of the polymer mass) were dispersed in 72.2 mL of THF in an ultrasonic bath for 6 h. Then, 2 g of PCL was added to the previous dispersion, left with constant stirring at RT for two hours, and finally poured into Petri dishes. PCL/G films (thickness between 120 and 140 µm) were obtained after solvent evaporation at RT for 72 h. The G nanosheets were expected to precipitate during solvent evaporation, forming a conductive surface layer of G nanosheets wrapped by the polymer matrix. The samples were finally vacuum-dried until constant weight to eliminate any residual solvent.

### 2.3. Morphological and Physico-Chemical Characterization of the Conductive Cell Substrates (PCL/G Nanocomposites)

The cell substrates morphology was analyzed by field emission scanning electron microscopy (FESEM) (Zeiss Ultra 55, Carl Zeiss Microscopy, Jena, Germany) with an accelerating voltage of 3 kV. Samples were coated with a platinum layer using a sputter coating (EM MED020, Leica, Wetzlar, Germany). The cross-section was observed by cryogenic fracture with liquid nitrogen. The morphology of the G nanosheets, previously dispersed in THF, was studied by high-resolution field-emission scanning electron microscopy (HRFSEM) (GeminiSEM 500, Carl Zeiss Microscopy, Germany) at acceleration voltages ranging between 0.7 and 1.5 kV.

Differential scanning calorimetry (DSC) (Perkin-Elmer DSC 8000, Perkin Elmer, Waltham, MA, USA) was performed under a flowing nitrogen atmosphere (20 mL/min) to analyze thermal behaviour. Samples were first heated up to 100 °C to erase the thermal history, followed by a cooling scan from 100 °C to −80 °C, ending with a second heating scan from –80 °C to 100 °C. All the scans were carried out at 20 °C/min. The glass transition temperature, *T_g_*, was calculated from the second heating scan as the inflexion point of the specific heat capacity, *C_p_*, vs. temperature, which coincides with a peak in the derivative heat capacity (*dC_p_*/*dT*).

The PCL degree of crystallinity was obtained as:

(1) $$X_{c}\left(\%\right)=\frac{\Delta H_{f}}{\Delta H_{f}^{0}}\cdot 100$$

where $\Delta H_{f}$ is the enthalpy of fusion of the samples and $\Delta H_{f}^{0}$ the enthalpy of fusion of totally crystalline PCL (139.5 J/g) [52].

The thermal stability and decomposition were analyzed by thermogravimetric analysis (TGA) (Mettler Toledo TGA 2 (SF), Mettler Toledo, Columbus, OH, USA). Vacuum-dried samples (≈5 mg mass) were heated from ambient temperature to 600 °C at a heating rate of 30 °C/min. The mass of the samples was recorded over the temperature range.

The mechanical properties of the substrates were studied by dynamic mechanical thermal analysis (DMA 8000, Perkin Elmer, Waltham, MA, USA) in sample bars (30 × 10 × 0.1 mm) operating in traction mode. The temperature dependence of the complex modulus E* (storage modulus (*E*′) and loss modulus (*E*″) was measured from −80 °C to 50 °C with a rate of 3 °C/min under a nitrogen atmosphere.

The electrical sheet resistance (*R_S_*) of neat PCL and PCL/G samples (10 mm diameter) was measured on a four-point probe system (T2001A3-EU, Ossila Ltd., Sheffield, UK). The electrical conductivity (*σ*) was obtained using the following expression:

(2) $$\sigma =\frac{1}{R_{S}\times l}$$

where *l* is the film thickness, obtained with a digital caliper (Acha, Eibar (Guipúzcoa), Spain). The measurements were carried out in triplicate to ensure reproducibility.

### 2.4. Biological Evaluation

#### 2.4.1. Cell Culture

Murine C2C12 myoblasts (Sigma-Aldrich-Merck, St. Louis, MO, USA) were expanded in a growth medium composed of high glucose Dulbecco’s Modified Eagle’s Medium (DMEM), (Biowest, Nuaillé, France) supplemented with 10% fetal bovine serum (FBS, ThermoFisher), and 1% penicillin/streptomycin (P/S) (ThermoFisher, Waltham, MA, USA) (growth medium) in 5% CO_2_ at 37 °C. Cells were passed during amplification upon reaching 80% confluence. All the experiments were performed on cells between six and eight passages. Four conditions were evaluated: non-conductive surfaces without additional extracellular Zn^2+^ ions (PCL condition), conductive nanocomposite surfaces (PCL/G) without extracellular Zn^2+^ (PCL/G condition), non-conductive surfaces with extracellular Zn^2+^ (PCL/Zn condition), and conductive nanocomposite surfaces with extracellular Zn^2+^ (PCL/G/Zn condition). ZnCl_2_ (concentration 40 µM), added to the culture medium, was used as the source of Zn^2+^ ions. This Zn^2+^ concentration was chosen from previous satisfactory results, in which this specific concentration was found to be suitable as myogenic factor for promoting myoblast proliferation and differentiation [40].

#### 2.4.2. Cytotoxicity

A direct MTS (3-[4,5, dimethylthiazol-2-yl]-5-[3-carboxymethoxy-phenyl]-2-[4-sulfophenyl]-2H-tetrazolium, inner salt) assay was performed to study the cytotoxicity of the engineered materials’ surface after 3 and 6 days.. This colorimetric assay is based on the reduction of MTS by viable and metabolically active cells into formazan dye in an enzymatic reaction carried out by NADPH-dehydrogenases. The formazan dye is quantified by measuring the absorbance at 490–500 nm.

Neat PCL and nanocomposites’ PCL/G cell substrates (three replicates with an area of 4 cm^2^) were first sterilized with three consecutive washings in 70% ethanol (10 min each) followed by a 30 min UV exposure for each sample surface. C2C12 at a density of 20,000 cells/cm^2^ were then cultured on the materials’ surface with growth medium (DMEM high glucose, 10% FBS, 1% P/S) in a humidified atmosphere at 37 °C and 5% CO_2_. Samples containing exogeneous Zn^2+^ (PCL/Zn and PCL/G/Zn) were inoculated into the culture medium with ZnCl_2_ 40 µM. After 3 and 6 days of culture (with medium renewal every 2 days), the growth medium was replaced by a medium without phenol red containing a 1:10 MTS dilution and left to incubate for 2 h to metabolize the MTS into formazan. The supernatant content of each well (three biological replicates per condition) was then transferred to a P96 plate (three technical replicates of each biological replicate), and absorbance at 490 nm was recorded using a fluorescence microplate reader (Victor Multilabel Plate Reader, Perkin Elmer, USA). Cell viability was calculated as:

(3) $$\mathrm{Viability}\;(\%)=\frac{OD\;test}{OD\;control\;}\cdot 100$$

where *OD test* is the optical density of the sample and *OD control* (life) is the optical density of the negative control (cells in growth media cultured on neat PCL substrates). Positive control (death) consisted of cells cultured in growth media with 2% Triton-X100 supplement, added 1 h before inoculating materials and controls with MTS.

#### 2.4.3. Cell Adhesion

Cell adhesion was evaluated in C2C12 myoblasts seeded at low density on the materials’ surface (5000 cells/cm^2^) to minimize cell contact. The materials (three replicates with 4 cm^2^ area) had been sterilized previously, as described in Section 2.4.2 and incubated overnight in growth media to ensure protein adsorption and surface functionalization. C2C12 myoblast were seeded on non-conductive substrates and conductive nanocomposites without additional extracellular Zn^2+^ (PCL and PCL/G conditions), and non-conductive and conductive cell substrates with Zn^2+^ (ZnCl_2_ 40 µM) in the culture medium (growth medium) (PCL/Zn, PCL/G/Zn conditions). After 24 h of culture at 37 °C and 5% CO_2_, the samples were fixed with 4% paraformaldehyde solution (1 h at RT), permeabilized with 0.5% Triton-X-100/DPBS, blocked with 5% horse serum (HS) in DPBS (1 h at 37 °C), and stained with fluorescent Phalloidin (dil:1:100, Thermo Fisher) to visualize the actin cytoskeleton. They were finally mounted with Vectashield containing DAPI (Palex Medical) and observed under a fluorescence microscope (Nikon Microscope Eclipse 80i). Cell area (actin cytoskeleton) was quantified on ImageJ software.

#### 2.4.4. Proliferation

C2C12 myoblasts were seeded at 10,000 cells/cm^2^ in previously sterilized and functionalized materials with 24 h incubation in growth medium to assess the proliferative effects of conductive cell surfaces and extracellular Zn^2+^ ions. We again assessed non-conductive substrates and conductive nanocomposites without extracellular Zn^2+^ ions (PCL and PCL/G conditions), and cell substrates supplemented with ZnCl_2_ 40 µM upon seeding and in each additional medium change (PCL/Zn and PCL/G/Zn conditions). After 1, 3 and 5 days of culture in growth medium to allow cells to grow and proliferate on the materials’ surface, the cells were fixed with paraformaldehyde solution (1 h at RT), permeabilized with 0.5% Triton-X-100/DPBS, blocked with 5% horse serum (HS) in DPBS (1 h at 37 °C), and mounted with Vectashield with DAPI to stain cell nuclei and quantify cell density after observation by fluorescence microscopy.

#### 2.4.5. Myogenic Differentiation

Myogenic differentiation was evaluated for the different conditions: non-conductive substrates and conductive surface nanocomposites with and without extracellular Zn^2+^ using two differentiation culture media. C2C12 were seeded at 20,000 cell/cm^2^ onto the surface of all sterilized materials previously incubated overnight in growth media (three replicates with area 4 cm^2^) for 24 h in growth medium. The culture medium was then changed to differentiation medium: DMEM supplemented with 2% FBS, 1% P/S (DMEM 2% FBS 1% P/S) as the first differentiation medium, and DMEM supplemented with 1% of insulin transferrin selenium (ITS, Sigma) and 1% P/S (DMEM 1% ITS 1% P/S) as the second differentiation medium. Again, PCL/Zn and PCL/G/Zn samples were supplemented with ZnCl_2_ 40 µM in the medium. The cells were cultured for 72 h, and then fixed with 4% paraformaldehyde (1 h at RT) and blocked with 5% HS in DPBS (1 h), permeabilized with 0.5% Triton-X-100 in DPBS and incubated with sarcomeric α-actinin antibody (Thermo Fisher, 1:200) (37 °C, 1 h), rinsed with DPBS, and incubated with secondary Alexa 488 antibody (Thermo Fisher, 1/500) (37 °C, 1 h). All samples were mounted with Vectashield mounting medium with DAPI and analyzed by fluorescence microscopy. Image quantification of myogenic differentiation was performed on ImageJ software.

The number of myotubes/cm^2^, mean myotube area, ratio of differentiated cells (number of nucleus inside differentiated cells/total nucleus), myotube diameter, average number of nuclei per myotube, and the ratio between multinucleated myotubes (≥2 nuclei)/mononucleated sarcomeric α-actinin positive cells area was used to quantify myoblast differentiation.

#### 2.4.6. Gene Expression Analysis (Real Time qPCR)

The expression of mTOR and MyoD-1 was analyzed after 3 days of cell culture following the procedure described in Section 2.4.5 using DMEM 2% FBS 1% P/S as differentiation medium. Total RNA was extracted by a RNeasy^®^ Mini Kit (Qiagen, Hilden, Germany) and quantified by the NanoDrop One microvolume UV/Vis Spectrophotometer system (Thermo Fisher). It was then reversed transcribed to cDNA (SimpliAmp thermal cycler, Applied Biosystems, Carlsbad, CA, USA) and RT-qPCR was performed by the SYBR Select Master Mix (Applied Biosystems) by a QuantStudio 5 Real-Time PCR System (Applied Biosystems). The expression of mTOR and MyoD-1 was analyzed, with GAPDH as the housekeeping gene. Data analysis was performed on QuantStudioTM software. The primers used for amplification were as follows: MyoD-1 (forward: 5′-GCA CTA CAG TGG CGA CTC AGA T-3′, reverse: 5′- TAG TAG GCG GTG TCG TAG CCA T-3′), mTOR (forward: 5′- AGA AGG GTC TCC AAG GAC GAC T-3, reverse: 5′- GCA GGA CAC AAA GGC AGC ATT G) and GAPDH (forward: 5′- CAT CAC TGC CAC CCA GAA GAC TG-3′, reverse: 5′- ATG CCA GTG AGC TTC CCG TTC AG-3′).

Quantification of gene expression was carried out by the comparative C_T_ method. Samples were normalized to the C_T_ value of the housekeeping gene (GAPDH): ΔC_T_ = C_T_ (sample) − C_T_ (GAPDH). C_T_ control values were those of the pristine PCL without extracellular zinc and/or graphene: ΔΔC_T_ = ΔC_T_ (sample) − ΔC_T_(PCL). mRNA expression was calculated according to the expression:

(4) $$\mathrm{Fold}\mathrm{change}=2^{\Delta \Delta C_{T}}$$

### 2.5. Statistics

The experiments were performed at least three times unless noted. All data are expressed as mean ± standard deviation. GraphPad Prism 8.0.2 software was used for statistical analysis. Differences between groups were analyzed by one-way analysis of variance (ANOVA tests). Statistical significance is indicated by (*) *p* < 0.05, (**) *p* < 0.01, and (***) *p* < 0.001.

## 3. Results

### 3.1. Morphology and Physico-Chemical Properties of PCL/G Nanocomposites

#### 3.1.1. Morphology

**Figure 1:**
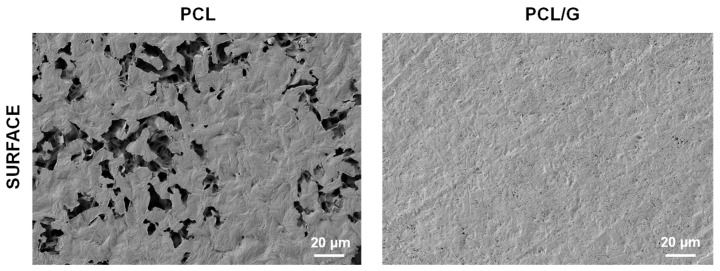
FESEM images of the surface of neat PCL and nanocomposites with 0.7 wt% of G nanosheets (PCL/G).

Figure 1 shows electron microscopy images of the surface of the PCL/G nanocomposite and pristine PCL substrates. The PCL surface shows a porous structure (with pore size up to 10 µm) formed during solvent evaporation and the formation of crystallites, which can also be seen in the cross-section (Supplementary Figure S1a). The layer containing PCL and G nanosheets, highly wrapped, can also be seen at the top of the cross-sectional image of the composite (Supplementary Figure S1a). Images of pristine G nanosheets after dispersion in THF in an ultrasonic bath show that they are around 3–4 µm long, tending to form small aggregates (Supplementary Figure S1b).

#### 3.1.2. Thermal Properties and Degradation

The thermal behavior of neat PCL (considered as reference) and PCL/G nanocomposites analyzed by DSC is shown in Supplementary Figure S2a. The melting process, between 45 and 60 °C, can be seen in the heating thermogram, while no cold crystallization took place during the heating. PCL glass transition emerged after applying the derivative, located ca. −60 °C. The experimental values of the glass transition temperature (*T_g_*) and the peak related to the melting process (*T_m_*) are given in Table 1. The addition of graphene nanosheets does not affect the PCL glass transition process (see the dotted area in the inset of Supplementary Figure S2a), although PCL crystallization is affected by its incorporation. The nanocomposites with 0.7% *wt*/*wt* of G nanosheets thus show slightly higher crystallinity, from 34.8% for pristine PCL to 38.3%.

The thermal decomposition of the nanocomposites, analyzed by TGA, are depicted in Supplementary Figure S2b (relative weight loss % and derivative of weight loss). The degradation profile of the nanocomposites is similar to that of pristine PCL, which degrades in a single weight loss step (320–480 °C).

#### 3.1.3. Mechanical and Electrical Properties

Dynamic mechanical thermal analysis (DMTA) was carried out between −80 and 50 °C to study the mechanical properties (Supplementary Figure S3 and Table 1). Both the storage modulus (*E*′) and loss modulus (*E*″) increased substantially after adding G nanosheets.

**Figure 2:**
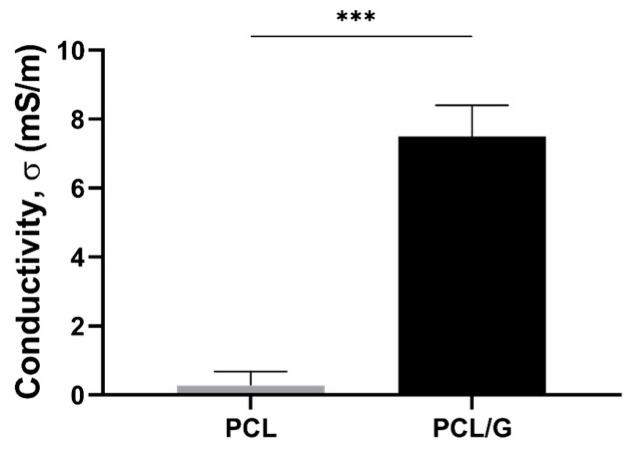
Electrical surface conductivity of PCL/G nanocomposites compared to pristine PCL substrate. (***) *p* < 0.001.

G nanosheets were introduced into the polymeric matrix to increase its surface electrical conductivity. The surface of PCL/G nanocomposites was prepared to obtain suitable electrical conductivity values for skeletal TE with a very small amount of G nanosheets to avert non-biocompatibility problems. The nanocomposite preparation procedure was designed to achieve a surface layer with large amount of G nanoparticles, which were darker in color, which considerably increased the surface conductivity of the PCL/G nanocomposite (7.5 ± 0.9 mS/m) (Figure 2), as reported elsewhere [53,54]. The biological assessment was performed with cells seeded on the conductive surface of the polymer nanocomposites.

### 3.2. Biological Performance In Vitro

Flat substrates of neat PCL and nanocomposite PCL/G were used as non-conductive and conductive surfaces, respectively (the morphology of the substrates and physico-chemical properties are included below). In addition, ZnCl_2_, as the source of Zn^2+^ ions, was added to the culture medium. Murine myoblasts C2C12 were chosen as the cell line to study the response in terms of adhesion, proliferation, and early myogenic differentiation. Four conditions were evaluated: non-conductive and conductive surfaces without exogeneous Zn^2+^ ions (PCL and PCL/G condition, respectively), and non-conductive and conductive surface with exogeneous Zn^2+^ ions (concentration: 40 µM) (PCL/Zn and PCL/G/Zn condition, respectively).

#### 3.2.1. Effect of a Conductive Surface and Exogeneous Zn Ions on Biocompatibility and Cell Adhesion

The biocompatibility of the substrates and the extracellular Zn^2+^ concentration added to the culture medium was assessed by an MTS assay in both non-conductive and conductive polymer nanocomposites (PCL/Zn and PCL/G/Zn conditions) by the direct method. The cells were seeded on the surface of the engineered substrates and Zn^2+^ ions (in the form of ZnCl_2_, 40 µM concentration) were added to the culture medium. Neat PCL substrates were used as reference (C-).

**Figure 3:**
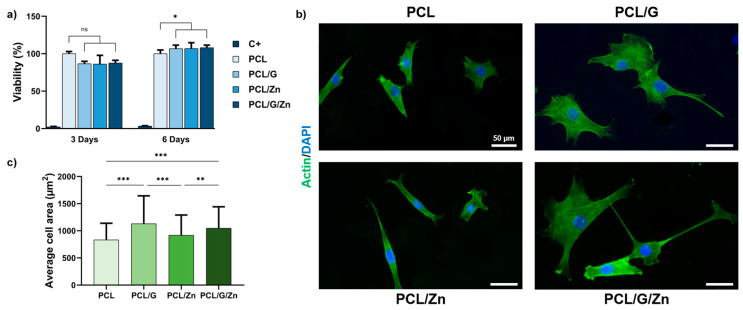
(**a**) MTS cytotoxicity. Cell viability after 3 and 6 days. Positive control: latex extract, negative control: neat PCL surface; (**b**) Immunofluorescence images of cell adhesion (actin staining); (**c**) Average cell adhesion area for the different conditions. (*) *p* < 0.05, (**) *p* < 0.01 and (***) *p* < 0.001.

Figure 3a shows the cytotoxicity after 3 and 6 days of culture. Cell viability less than 70% of the positive control is considered cytotoxic (ISO standard 109935). The G-based nanocomposites (PCL/G), as well as the addition of 40 µM of Zn^2+^ in the culture medium (PCL/Zn condition), show viability values of over 80% in both evaluations, with no significant differences from the PCL substrates (considered as 100% viability). Combining the polymeric matric with 0.7% *wt*/*wt* of G nanosheets as cell surface and 40 µM of exogeneous Zn^2+^ (PCL/G/Zn condition), the viability is still above 80%.

Cell adhesion was analyzed after 24 h of culture on samples pre-conditioned with protein adsorbed on the material surfaces (Figure 3b,c). Actin staining showed a significant increase in average cell area (>13%) in G nanocomposites (PCL/G) compared to neat PCL substrates (considered as reference). Conversely, PCL substrates with exogenous Zn^2+^ ions (PCL/Zn) did not show a significant enhancement of cell adhesion. Finally, the combination of conductive cell substrate and exogeneous Zn^2+^ ions (PCL/G/Zn) results in similar cell spreading to the conductive nanocomposite (PCL/G).

#### 3.2.2. Effect of a Conductive Surface and Exogeneous Zn Ions on Myoblast Proliferation and Differentiation

**Figure 4:**
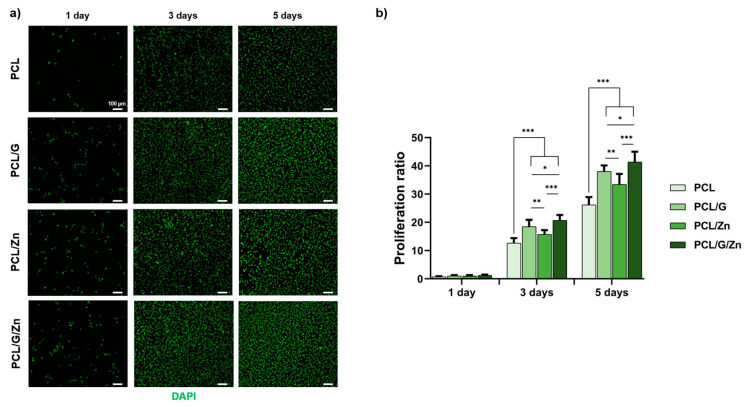
Myoblast proliferation seeding in growth medium. (**a**) Representative cell density (DAPI nuclei staining) at 1, 3 and 5 days after culture; (**b**) Proliferation ratio obtained as the ratio between total cell density and initial cell density. (*) *p* < 0.05, (**) *p* < 0.01, and (***) *p* < 0.001.

Cell proliferation experiments were performed on myoblasts seeded onto the surface of non-conductive PCL substrates and conductive PCL/G nanocomposites with and without extracellular Zn^2+^ ions (concentration: 40 µM) to investigate the potential mitogenic effect of the combined strategy proposed in this study. Proliferation ratio (cell density/initial cell density) was analyzed after 1, 3 and 5 days of culture (Figure 4). No significant differences were found after 1 day of culture, although the conductive PCL/G substrates (without extracellular Zn^2+^) and PCL substrates with extracellular Zn^2+^ significantly increased in cell density with respect to pristine PCL after 3 and 5 days of culture. Finally, the combination of the conductive properties of the substrate and the presence of extracellular Zn^2+^ ions (PCL/G/Zn condition) was found to significantly enhance cell proliferation, compared to both conditions applied separately.

**Figure 5:**
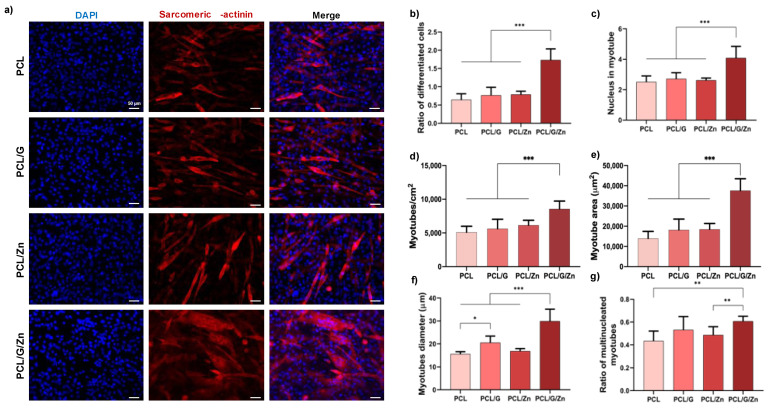
Myoblast differentiation (DMEM +2%FBS +1% P/S culture medium). (**a**) Immunofluorescence images of differentiated myoblasts (sarcomeric α-actinin staining) after 3 days of culture; (**b**) Ratio of differentiated cells relative to initial cell density; (**c**) Average number of nuclei inside myotubes; (**d**) Myotube density; (**e**) Myotube area; (**f**) Myotube diameter analysis of 80 random myotubes per condition; (**g**) Ratio of multinucleated myotubes. (*) *p* < 0.05, (**) *p* < 0.01, and (***) *p* < 0.001.

To investigate the synergies of a conductive nanocomposite surface and exogeneous Zn^2+^ ions (40 µM concentration) in inducing myoblast differentiation in the early stages of commitment to differentiation, C2C12 cells were first cultured in DMEM supplemented with 2% of FBS (serum-deprived medium), a conventional way of inducing myoblast differentiation [23]. Figure 5a shows immunofluorescence images and quantification of myoblast differentiation after 3 days of culture on non-conductive substrates and conductive substrates, with and without exogeneous Zn^2+^. Conductive PCL/G nanocomposites (without extracellular Zn^2+^), and non-conductive substrates with extracellular Zn^2+^ (PCL/Zn condition) showed increased values for several parameters related to myogenic differentiation.

The combination of a conductive nanocomposite surface and exogeneous Zn^2+^ ions (PCL/G/Zn condition) provided an outstanding result. The ratio of differentiated cells was around 170% higher than that of neat PCL (from 0.64 to 1.73), with an increase of ca. 126% and 120% over PCL/G and PCL/Zn conditions, respectively. Regarding the average number of nuclei per myotube (Figure 5c), it was found that the combined strategy substantially raised the number of fused cells, while an enhancement close to 70% was found between the combination of both effects and neat PCL substrates with regard to the number of myotubes/cm^2^ (Figure 3d). The average myotube area and diameter (Figure 5e,f) were also significantly enhanced in PCL/G/Zn condition over all other conditions. The mean myotube diameter (Figure 5f) was higher in the conductive substrates (PCL/G) than in neat PCL (from 15.5 to 20.5 µm). Again, the combination of a conductive surface and exogeneous Zn^2+^ significantly raised the average myotube diameter to ca. 30 µm (more than 90% higher than the average myotube diameter on pristine PCL substrates). Finally, the combination of a conductive nanocomposite surface and extracellular Zn^2+^ ions (PCL/G/Zn condition) (Figure 5g) significantly increased the ratio of multinucleated myotubes compared to neat PCL (ca. 40%), and PCL/Zn (ca. 25%).

**Figure 6:**
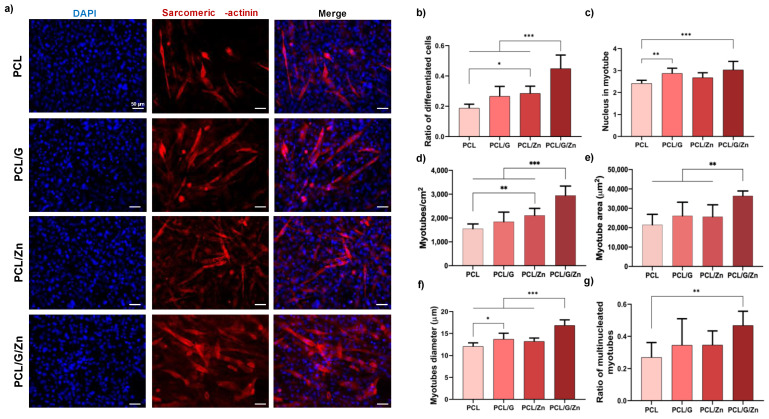
Myoblast differentiation in differentiation medium (DMEM +1% ITS +1% P/S. (**a**) Immunofluorescence images of differentiated myoblasts (sarcomeric α-actinin staining) after 3 days of culture; (**b**) Ratio of differentiated cells relative to initial cell density; (**c**) Average number of nuclei inside myotubes; (**d**) Myotube density; (**e**) Myotube area; (**f**) Myotube diameter analysis of 80 random myotubes per condition; (**g**) Ratio of multinucleated myotubes. Graphs show mean ± standard deviation. (*), (**) and (***) indicate significant differences (*p* < 0.05, *p* < 0.01, and *p* < 0.001, respectively).

Similar results were obtained in C2C12 myoblasts cultured in DMEM supplemented with 1% of insulin-transferrin-selenium (ITS) as differentiation medium (Figure 6). The differentiated cell ratio of conductive substrates supplemented with extracellular Zn^2+^ was ca. 140% higher than that of non-conductive PCL, and 69% and 55% higher than PCL/G and PCL/Zn conditions, respectively. In addition, the combination of a conductive surface and exogeneous Zn^2+^ ions significantly increased the number of nuclei inside myotubes, number of myotubes/cm^2^, myotube area, myotube diameter, and the ratio of multinucleated myotubes over those of all the other conditions.

## 4. Discussion

Our aim was to analyze the synergies between an electrically conductive biomaterial surface (polymeric matrix and G nanosheets as conductive filler) and extracellular Zn^2+^ ions as bioactive factor to stimulate myoblast response.

Nanocomposites based on a biodegradable polymer matrix (PCL) and G nanosheets were first prepared to obtain flat cell substrates with surface conductive properties suitable for muscle-skeletal TE. As stated previously, PCL-based polymers have been used as biomaterials to develop numerous strategies to generate skeletal muscle tissues in vitro and, in this study, was used as reference cell substrate in the biological evaluation. Classical solvent casting was a suitable method to prepare the flat cell substrates with conductive surface properties. Thus, highly diluted PCL-THF solutions were used in the manufacturing process (3% *wt*/*wt* PCL/THF) incorporating G nanosheets at a concentration of 0.7% *wt*/*wt* (with respect to the mass of PCL). The diluted solution favours the deposition of the highly conductive G nanosheets on the bottom of the Petri dish during solvent evaporation, forming a superficial layer of G nanosheets wrapped by the polymer chains. As expected, after solvent evaporation, a darker coloration was observed on the surface in contact with the Petri dish, which indicated the presence of a higher amount of G nanosheets highly embedded within the polymeric matrix. Adding G nanosheets significantly changed the morphology, so that the PCL/G nanocomposites had a smooth surface without pores, which suggests the presence of G nanosheets precipitated during solvent evaporation, forming a uniform layer of PCL/nanoparticles.

The DSC thermogram of the PCL/G nanocomposites (Figure S2a and Table 1) showed that the crystallization of PCL increases after the addition of G nanosheets, indicating that the G nanosheets act as a nucleating agent, in good agreement with previous results [55]. However, the thermal stability of the polymer matrix, shown in Figure S2b, is not substantially affected by adding very small amounts of G nanosheets [56]. As expected, the mechanical properties increased in the nanocomposite compared to the neat PCL substrates, in agreement with previous results, which showed that a low G content can improve the mechanical properties [57,58]. This increase can be attributed to the intrinsic properties of the filler, i.e., the dispersion and distribution of the G nanosheets embedded within the polymeric matrix [59]. At 37 °C, the storage modulus of the nanocomposite is more than double that of the pristine PCL (Table 1). However, the improvement obtained is lower than that reported in other studies with composites containing carbon nanomaterials [54,60], which could indicate the presence of G aggregates within the sample [61]. This result is consistent with the aim of our study, in which the nanocomposite preparation procedure used allows obtaining a greater amount of G nanosheets on the surface. Thus, the surface conductivity in the smooth face of the PCL/G nanocomposite (Figure 2), where the G nanosheets precipitated, is in the range of other reported electroactive biomaterials that have stimulated cell response [28,62], and particularly close to that of natural human skeletal muscle (0.8 × 10^−3^~4.5 × 10^−3^ S/m) [63]. The biological evaluation was carried out using this conductive smooth surface of the nanocomposite as cell substrate.

The biological assessment was performed in mouse myoblast cell line (C2C12), a well-established model to study myogenesis in vitro [64], with conductive and non-conductive substrates, and with and without the addition of Zn^2+^ ions in the culture medium. Previous results had shown that exogeneous Zn^2+^ ions, in particular, 40 µM of Zn^2+^ added to culture medium, enhanced myoblast proliferation and differentiation [40], which was why we used this specific Zn^2+^ concentration as exogeneous bioactive factor.

The biocompatibility of PCL is well known [47], although the cytotoxicity of biomaterials that include carbon nanomaterials still has to be verified. Both the concentration and the method used to prepare the biomaterial have been reported to be directly related to the performance in biological environments [27]. The biocompatibility assay (Figure 3a) showed that neither the conductive polymer nanocomposites, the addition of exogenous Zn at 40 µM in the culture medium, nor the cellular microenvironment created by the combined strategy is cytotoxic.

Cell adhesion on PCL/G nanocomposites increased compared to neat PCL substrates (Figure 3b,c). Due to its high specific surface area, graphene possesses a large capacity for protein absorption. It is well known that the protein coating induces changes in surface charges, hydrophilicity or topography, affecting the bioactivity [65]. The enhancement in cell attachment on PCL/G nanocomposites, specifically the cell area, may be attributed to an increase in the adhesion of absorption proteins, such as fibronectin and vinculin [40,66]. In the other hand, neat PCL with Zn^2+^ ions added in the culture medium had no effect on cell adhesion. It has been reported that Zn^2+^ provides a positive effect on cell adhesion, depending on its content and cell line. Cell adhesion correlates with the concentration of Zn^2+^ ions, and small amount of Zn may not enhance cell adhesion [40]. These results suggest that the small amount of extracellular Zn^2+^ used in this study (40 µM) is not enough to increase cell adhesion. In line with the above, the combination of conductive substrates and extracellular Zn^+2^ has no impact on myoblast adhesion.

The significant increase of proliferation after 3 and 5 days of cell culture on PCL/G substrates (Figure 4) is in line with previous results that demonstrated that cell substrates containing conductive materials, such as conductive polymers, carbon-based nanomaterials or metal-based nanomaterials, induced proliferation in several cell lines [67,68,69]. Likewise, an increase in cell proliferation was expected after the addition of extracellular Zn^2+^ in PCL substrates (PCL/Zn condition), as reported elsewhere [40]. The significant increase in myoblast proliferation after the addition of extracellular Zn^2+^ on cells seeded on conductive substrates (PCL/G/Zn condition) with respect to both conditions separately, confirms the synergistic mitogenic effect of the combined strategy. This finding indicates that the molecular mechanism involved in myoblast proliferation is stimulated by the cell microenvironment created by the conductive substrates and the extracellular Zn^2+^.

The synergistic effect of the combined strategy was assessed in C2C12 cultured in two differentiation media (serum-deprived medium and DMEM+ITS) after 3 days of culture (Figure 5 and Figure 6). Conductive substrates and non-conductive substrates with extracellular Zn^2+^ showed enhanced behavior in some parameters of myogenic differentiation in both culture media. However, as expected since it is a 3-day cell culture (early differentiation stage), the effect of the incorporation of G nanosheets and extracellular Zn^2+^ ions was therefore lower than that obtained in previous studies (with longer culture time), in which both G [23,29] and exogeneous Zn^2+^ [40] were shown to promote myogenic differentiation. Interestingly, a substantial increase was found by the combined strategy, with significant differences in most of the characteristic parameters with respect to all the other conditions, regardless of the differentiation medium used for the cell culture. These results confirm that the synergistic effect of the conductive surface and extracellular zinc promote the early stages of myogenic differentiation.

**Figure 7:**
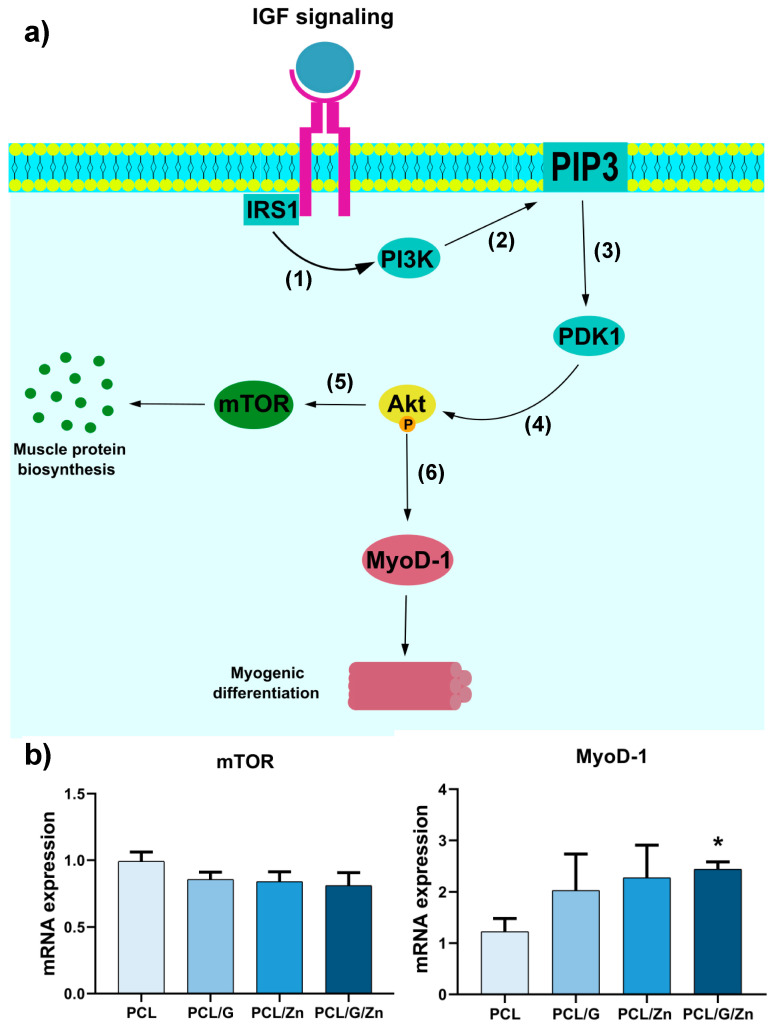
(**a**) Representative scheme of the simplified IGF/PI3K/Akt signaling pathway and its enrolment in muscle growth and myogenic differentiation. (1), (2) reference [40]; (3), (4) references [40,81]; (5) reference [73]; (6) reference [74]; (**b**) Gene expression analysis results (RT-qPCR) for mTOR and MyoD-1 after culture in serum-deprived medium (DMEM +2% FBS +1% P/S) with GAPDH as housekeeping gene. (*) *p* < 0.05 between PCL and PCL/G/Zn conditions.

To further analyze the effect of the combined proposed strategy, gene expression analysis was performed by RT-qPCR to evaluate regulatory factors mTOR and MyoD-1, both related to the widely studied IGF/PI3K/Akt pathway, albeit with different functions [40,70]. The activation (phosphorylation) of protein kinase Akt-1 by the IGF/PI3K signaling cascade has been reported to lead to an increase in the activity of mTOR protein kinase, related to myogenic protein biosynthesis and cell growth, survival, and proliferation [71,72,73]. In vitro and in vivo experiments showed that mTOR activity not only promotes cell proliferation, but also inhibits myogenic differentiation in muscle development [73]. In addition, several studies have proven that Akt-1 activation by IGF/PI3K cascade increases the activity of MyoD-1, a transcriptional factor involved in the early stages of commitment to myogenic differentiation, establishing a signaling cascade that induces cell cycle arrest, a prerequisite for myogenesis initiation [40,70,74,75,76]. Figure 7a depicts the chain of events following the IGF/PI3K/Akt signaling pathway leading to mTOR expression, related to muscle protein expression (cell growth/proliferation), and MyoD-1 expression (involved in the early stages of myogenic differentiation).

The qPCR results, shown in Figure 7b, are consistent with those reported previously, as the cells cultured in differentiation medium (serum-deprived medium in this assay) did not produce significant differences in mTOR expression in any of the condition tested. Neither PCL/G, PCL/Zn or PCL/G/Zn showed significant differences compared to PCL, indicating no proliferative activity induced by conductive substrates or extracellular Zn^2+^ [73]. Nevertheless, MyoD-1 expression behaved differently, with a remarkable increase in PCL/G/Z condition with respect to neat PCL substrates. Thus, MyoD-1 expression in PCL/G/Zn condition was twice that of neat PCL substrates, while PCL/G and PCL/Zn conditions showed no significant differences in MyoD-1 expression compared to neat PCL.

PI3K/Akt is well known as an essential pathway in proliferation, differentiation, and cell survival, through the activation of Akt protein [77]. Neuronal differentiation of bone marrow mesenchymal stem cells (BMSCs) has been reported to be promoted by the electrical properties of a collagen/hyaluronan hydrogel with conductive PPy nanoparticles and electrical stimulation, which is related to the PI3K/Akt signaling pathway [78]. Liu et al. showed that the PI3K/Akt pathway is involved in cell growth induced by conducive graphene in representative cell lines (HepG2, A549, MCF-7 and HeLa) [67]. Graphene has also been suggested to promote stem cell differentiation through the activation of PI3/Akt signaling pathway [77], while several studies have indicated the important role of Zn ions in this signaling pathway [79]. In fact, extracellular Zn^2+^ ions have been shown to stimulate myoblast response via the PI3K/Akt signaling cascade [40,80]. The results obtained suggest that the combined strategy applied in the present study (cell–substrate interface with conductive properties and extracellular Zn^2+^) synergically stimulate PI3K/Akt, which result in an early commitment to myoblast differentiation. Further analyses will be necessary to reveal the cell mechanisms triggered by this combined strategy, including the analysis of myoblast differentiation for longer periods of time (later stages of myogenic differentiation) and the role of the combined strategy on specific signaling pathways, as well as the effect of external electrostimulation.

## 5. Conclusions

In this study, we developed new cell environments based on the combination of a 2D conductive polymer nanocomposite and extracellular Zn ions as myogenic factor. The conductive nanocomposite surface was prepared from a polymeric matrix and a small percentage of graphene nanosheets (0.7% *wt*/*wt*) to evaluate the effect of this synergistic approach on myoblast adhesion, proliferation, and differentiation when combined with extracellular Zn^2+^ ions (concentration 40 µM). PCL/G nanocomposites showed a smooth surface with no porosity. As expected, electrical properties of the cell substrates significantly increased in terms of surface conductivity, after graphene nanosheets addition, with conductivity in the range of biological skeletal muscle tissue. Biocompatibility was confirmed in murine C2C12 myoblasts after 3 and 6 days of culture. The novel cell microenvironment showed good cell adhesion and spreading. A strong synergistic effect induced by the combined strategy was found on myoblast proliferation and cell differentiation (performed in two differentiation media), in which early myoblast differentiation was induced by this novel strategy. However, further studies are required to optimize the system by varying the conductive properties of the substrates and the concentration of mount of extracellular Zn^2+^. From those results, 3D substrates will be engineered, using electrospinning or 3D printing as potential techniques, including both conductive surfaces and the release of Zn ions previously loaded in the substrate. In conclusion, considering the in vitro results obtained in this study, this straightforward and efficient strategy combining a conductive surface and extracellular zinc ions shows great potential for applications in skeletal muscle tissue engineering.

## Figures and Tables

**Table 1:** Glass transition temperature (T_g_), melting temperature (T_m_), enthalpy of fusion (∆H_f_), degree of crystallinity (X_c_ %) and storage modulus (E’) at 37 °C.

Sample	T_g_ (°C)	T_m_ (°C)	ΔH_f_ (J/g)	X_c_ (%)	E’ (Pa) at 37 °C
PCL	−64.8	53.8	48.5	34.8	3.9·10^8^
PCL/G	−64.4	55.2	53.1	38.3	8.2·10^8^

## Data Availability

Data will be made available on request.

## Supplementary Materials

The following supporting information can be downloaded at: https://www.mdpi.com/article/10.3390/biology11121706/s1, Figure S1: (**a**) Cross-section of neat PCL and nanocomposites with 0.7 wt% of G nanosheets (PCL/G). (**b**) HRFESEM representative images of pristine graphene nanosheets (aggregated and single form) previously dispersed in THF; Figure S2: (**a**) Differential scanning calorimetry (DSC) thermograms of neat PCL and PCL/G nanocomposites. Normalized heat flow (Cp) (2nd scan, heating). Inset: temperature derivative of the heat capacity (dCp/dT) from −70 to 0 °C; the dotted lines indicate the glass transition process. (**b**) Thermogravimetry results (TGA). Relative weight loss of PCL and PCL/G nanocomposites; Figure S3: Dynamic mechanical thermal analysis (DMTA traction assay). *E*′ and *E*″ vs. temperature at 1 Hz.
