# Supplementary material for: Pro-Myogenic Environment Promoted by the Synergistic Effect of Conductive Polymer Nanocomposites Combined with Extracellular Zinc Ions
Source: Biology (Basel). 2022 Nov 25;11(12):1706. doi: 10.3390/biology11121706 (PMC9774464; doi:10.3390/biology11121706)
Supplement: Supplementary file 1 [file biology-11-01706-s001.zip › biology-2030835-supplementary.pdf]

Pro-myogenic environment promoted by the synergistic effect of conductive polymer nanocomposites combined with extracellular zinc ions

J.L. Aparicio-Collado[1], J. Molina-Mateo[1], C. Torregrosa Cabanilles[1], A. Vidaurre[1,3], Beatriz Salesa[4], A. Serrano-Aroca[4,*], R. Sabater i Serra[1,2,3,*]

[1] Centre for Biomaterials and Tissue Engineering, Universitat Politècnica de València, 46022 Valencia, Spain

[2]Department of Electrical Engineering, Universitat Politècnica de València, 46022 Valencia, Spain

[3]Biomedical Research Networking Centre in Bioengineering, Biomaterials and Nanomedicine (CI-BER-BBN), 46022 Valencia, Spain

[4] Biomaterials and Bioengineering Lab, Centro de Investigación Traslacional San Alberto Magno, Universidad Católica de Valencia San Vicente Mártir, 46001 Valencia, Spain

***Correspondence:** Roser Sabater i Serra (rsabater@die.upv.es) and Ángel Serrano-Aroca (angel.serrano@ucv.es)

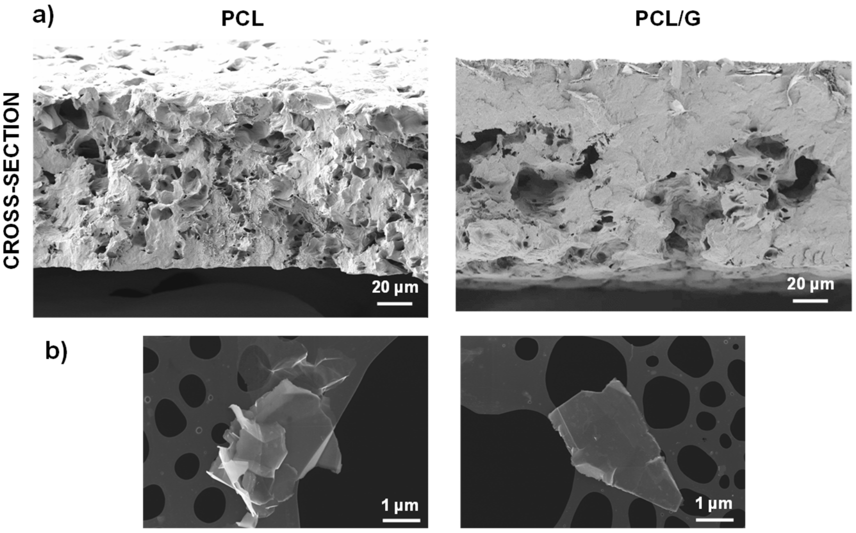

Figure S1. (**a**) Cross-section of neat PCL and nanocomposites with 0.7 wt% of G nanosheets (PCL/G). (**b**) HRFESEM representative images of pristine graphene nanosheets (aggregated and single form) previously dispersed in THF.

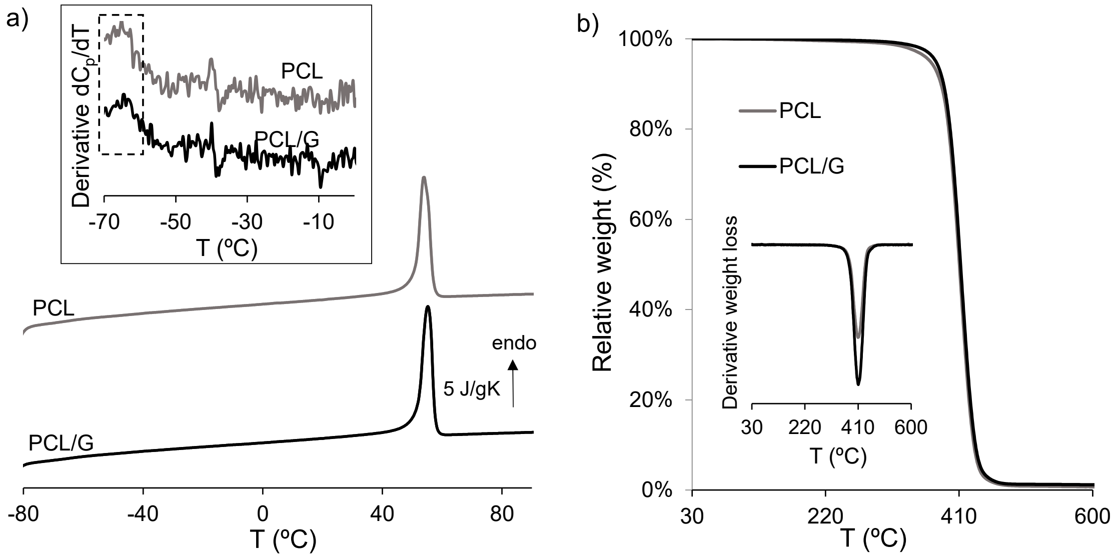

Figure S2. (**a**) Differential scanning calorimetry (DSC) thermograms of neat PCL and PCL/G nanocomposites. Normalized heat flow (C_p) (2nd scan, heating). Inset: temperature derivative of the heat capacity (dC_p/dT) from -70 to 0 ºC; the dotted lines indicate the glass transition process. (**b**) Thermogravimetry results (TGA). Relative weight loss of PCL and PCL/G nanocomposites. Inset: derivative of weight loss.

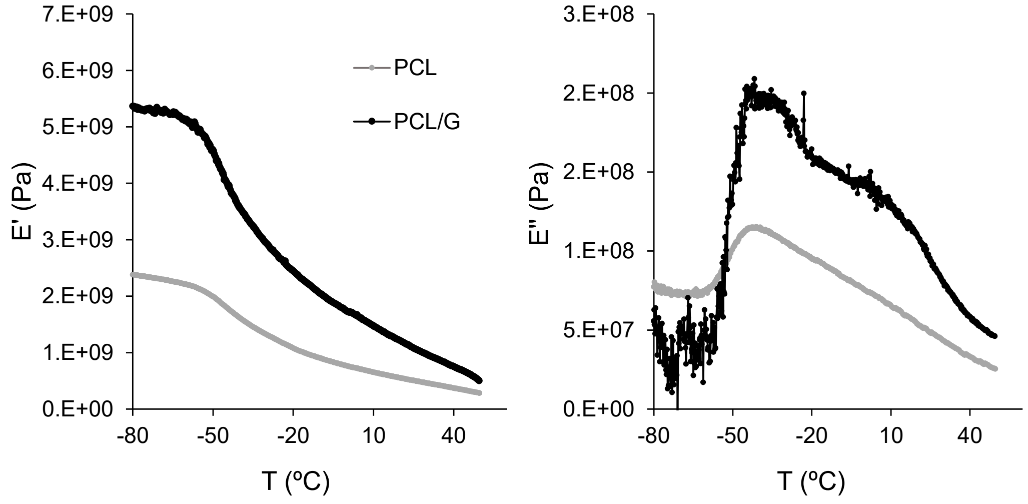

Figure S3. Dynamic mechanical thermal analysis (DMTA traction assay). *E'* and *E''* vs. temperature at 1 Hz.
